# Nano‐Metamaterial: A State‐of‐the‐Art Material for Magnetic Resonance Imaging

**DOI:** 10.1002/smsc.202300015

**Published:** 2023-07-04

**Authors:** Qiyue Wang, Hui Du, Fangyuan Li, Daishun Ling

**Affiliations:** ^1^ Frontiers Science Center for Transformative Molecules School of Chemistry and Chemical Engineering National Center for Translational Medicine State Key Laboratory of Oncogenes and Related Genes Shanghai Jiao Tong University Shanghai 200240 P. R. China; ^2^ World Laureates Association (WLA) Laboratories Shanghai 201203 P. R. China; ^3^ Institute of Pharmaceutics College of Pharmaceutical Sciences Zhejiang University Hangzhou 310058 P. R. China; ^4^ Hangzhou Institute of Innovative Medicine Zhejiang University Hangzhou 310058 P. R. China

**Keywords:** contrast agents, metamaterials, magnetic resonance imaging, multilevel microarchitectures

## Abstract

Metamaterials are artificially designed materials with multilevel‐ordered microarchitectures, which exhibit extraordinary properties not occurring in nature, and their applications have been widely exploited in various research fields. However, the progress of metamaterials for biomedical applications is relatively slow, largely due to the limitations in the size tailoring. When reducing the maximum size of metamaterials to nanometer scale, their multilevel‐ordered microarchitectures are expected to obtain superior functions beyond conventional nanomaterials with single‐level microarchitectures, which will be a prospective candidate for the next‐generation diagnostic and/or therapeutic agents. Here, a forward‐looking discussion on the superiority of nano‐metamaterials for magnetic resonance imaging (MRI) according to the imaging principles, which is attributed to the unique periodic arrangement of internal multilevel structural units in nano‐metamaterials, is presented. Moreover, recent advances in the development of nano‐metamaterials for high‐performance MRI are introduced. Finally, the challenges and future perspectives of nano‐metamaterials as promising MRI contrast agents for biomedical applications are briefly commented.

## Graphical abstract

Herein, the superiority of magnetic nano‐metamaterials for MRI is highlighted according to the imaging principles. The recent advances, current challenges, and future development of nano‐metamaterials as MRI contrast agents for biomedical applications are commented. The ingeniously designed nano‐metamaterials shall greatly promote groundbreaking medical diagnosis research and elucidate unknown life phenomena.

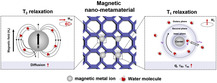

## 1 Introduction

**Figure 1:**
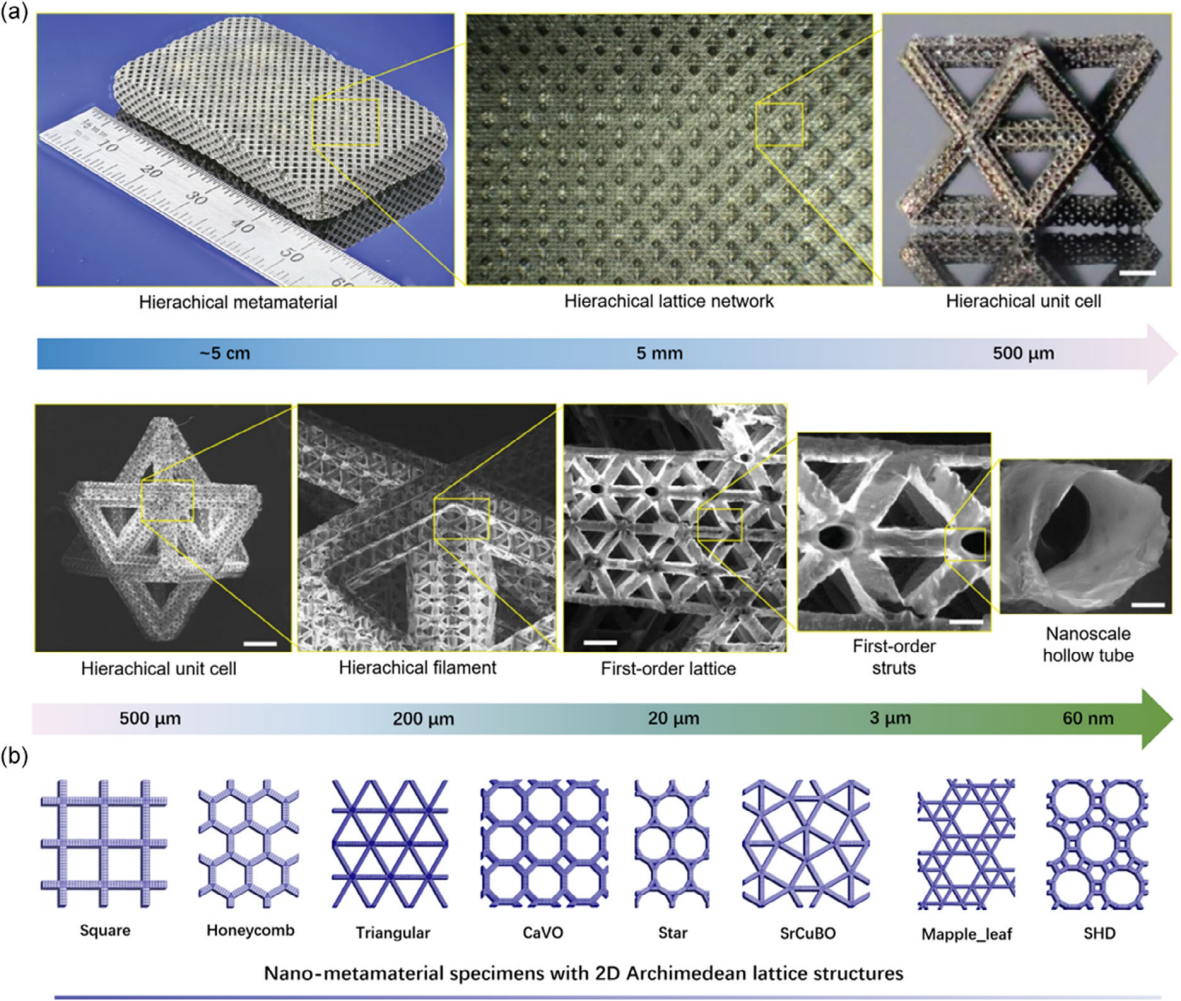
a) The critical features of nickel alloy layered metamaterials across seven orders of magnitude in length scale. Reproduced with permission.^[^ ^2^ ^]^ Copyright 2016, Springer Nature. b) Schematic illustration of a ferroelectric nano‐metamaterial with a 2D Archimedean lattice structure. Reproduced under the terms of the CC‐BY Creative Commons Attribution 4.0 International license (https://creativecommons.org/licenses/by/4.0).^[^ ^11^ ^]^ Copyright 2015, The Authors, published by Springer Nature.

Metamaterials are artificially designed hierarchical materials with multilevel‐ordered microarchitectures, which exhibit remarkable characteristics not occurring in nature. Their unique properties and functionalities are primarily derived from the ingenious design of artificial structural units with a periodic arrangement and precise control of the key parameters.^[^ ^1^, ^2^ ^]^ In contrast to conventional materials with single‐level microarchitectures, metamaterials possess multilevel complexity by integrating architectural and compositional diversity into a single superstructure (**Figure** **1**a).^[^ ^3^, ^4^ ^]^ This feature endows metamaterials with extraordinary properties and functionalities that transcend the simple combination of their components. Over the past few decades, the development of metamaterials can be summarized into several phases. It was first proposed by Veselago in 1968 with concepts and fundamental theorems focused on the negative‐index metamaterials in the microwave region.^[^ ^5^ ^]^ Subsequently, such a deceptively simple but extraordinarily powerful concept was rapidly extended to a much broader range in the field of electromagnetism, optics, acoustic, and mechanics, and displayed highly distinctive properties, such as ultrahigh positive refractive index,^[^ ^6^ ^]^ enhanced nonlinear optical properties,^[^ ^7^ ^]^ as well as reprogrammable mechanical stiffness.^[^ ^8^, ^9^ ^]^

Despite significant advances in the fundamental research of metamaterials, their practical applications in certain fields are still hampered by the limitations of size tailoring, especially in the nanomedicine field where the maximum size is required to be tens to hundreds of nanometers.^[^ ^10^ ^]^ It has been demonstrated that metamaterials can be manufactured down to the nanometer level (Figure 1b),^[^ ^11^ ^]^ which shows great promise to expand their potential applications, particularly in the biomedical field. Nevertheless, how to utilize state‐of‐the‐art nanofabrication technologies to achieve the precise design and synthesis of complicated nano‐metamaterial with functions beyond that possible by conventional nanofabrication and engineering tools, still remain challenging and will be the thematic issue of the next development stage of metamaterials.

**Figure 2:**
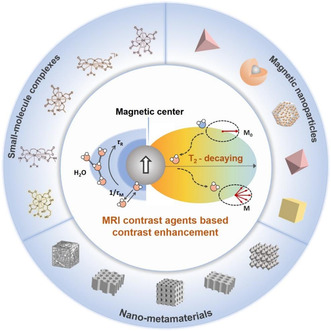
Schematic illustration of the structure of conventional MRI contrast agents (paramagnetic small‐molecule complexes and magnetic nanoparticles with single‐level microarchitectures) and nano‐metamaterials. Owing to the unique multilevel microarchitectures, nano‐metamaterials hold great potential to enhance the important parameters affecting the relaxation times of water protons and thus carve out a new horizon in the field of MRI.

For biological applications, the development of cutting‐edge materials employed as contrast agents in the field of medical imaging is highly desired. Currently, various tailor‐made materials for enhancing contrast in medical imaging (e.g., computed tomography, positron emission computed tomography, magnetic resonance imaging (MRI), etc.) have played an important role in preclinical and clinical research.^[^ ^12^, ^13^, ^14^, ^15^, ^16^ ^]^ Among these, MRI is a noninvasive and radiation‐free imaging technique with excellent soft tissue resolution, which is widely applied in clinical diagnosis, such as various malignant lesions, tissue necrosis, and local ischemia.^[^ ^17^ ^]^ The utilization of MRI contrast agents can accelerate the longitudinal (*T*_1_) or transverse (*T*_2_) relaxation of surrounding water protons, thus improving the signal‐to‐noise ratio and imaging contrast.^[^ ^18^ ^]^ The structure of contrast agents determines the imaging capability,^[^ ^19^ ^]^ and thus the development of high‐performance MRI contrast agents via exquisite structural regulation is helpful to achieve accurate diagnosis of major diseases even at the early stage.^[^ ^20^ ^]^ Compared with conventional contrast agents, including paramagnetic small‐molecule complexes and simple magnetic nanoparticles with single‐level microarchitectures,^[^ ^21^ ^]^ the magnetic nano‐metamaterials exhibit unique structural properties of ordered multilevel multiscale periodically arranged microarchitectures, which necessarily possess extraordinary imaging performance and could carve out a new horizon in the field of MRI (**Figure** **2**). In this Perspective, the unique advantages of nano‐metamaterials for MRI are discussed, as well as their recent advances and future developments are commented.

## 2 Superiority of Nano‐Metamaterial for MRI

**Figure 3:**
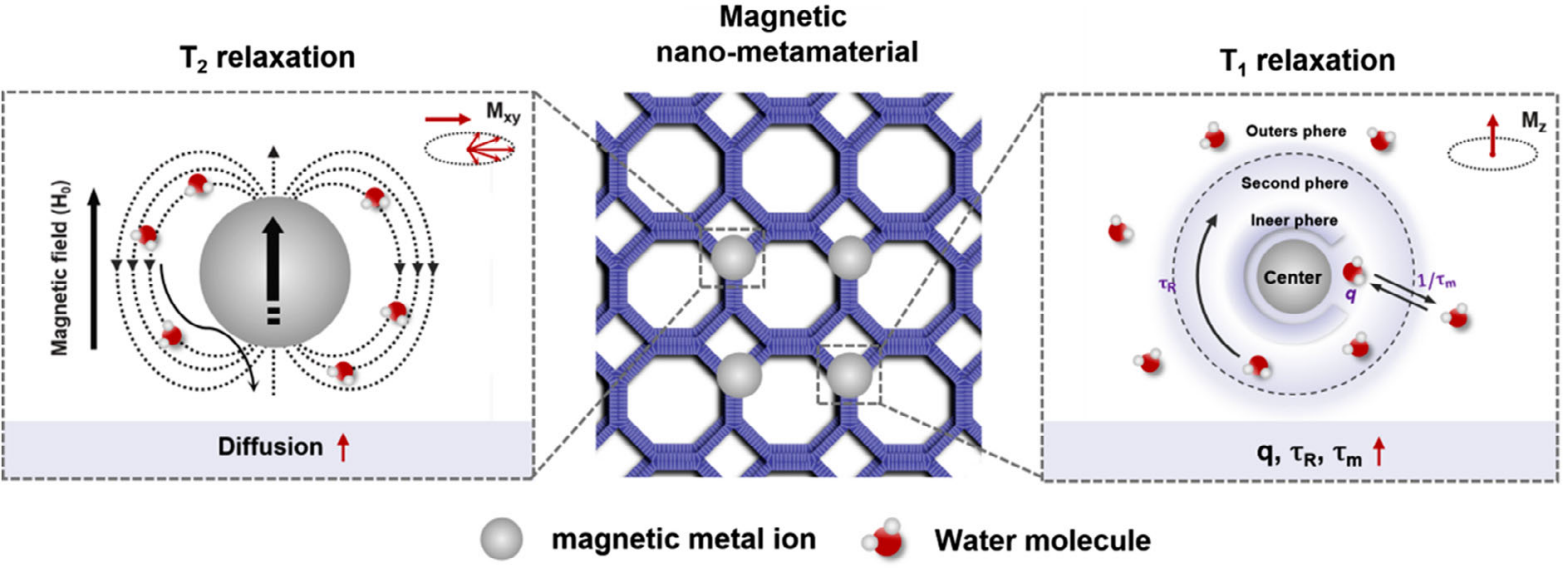
Schematic illustration of the mechanism for the enhanced *T*_2_ and *T*_1_ relaxation of water protons in magnetic nano‐metamaterials. Regarding *T*_2_ relaxation rates, the dense multilevel periodically arranged microarchitectures of nano‐metamaterials contribute to a significant enhancement of the overall magnetization, and thus affecting the diffusion rate of water protons. Besides, the unique structural features of nano‐metamaterials can promote the interaction of water molecules with the internal paramagnetic ions, and trap water molecules in the interstices of their complicated microarchitectures, which lead to larger *q*, longer $\tau_{m},$and $\tau_{\text{R }}$ than those of conventional nanoparticulate contrast agents, thus accelerating the *T*_1_ relaxation of water protons.

Most clinically available MRI contrast agents are paramagnetic gadolinium ionic (Gd^3+^) complexes, which produce positive *T*_1_ contrast and brighten the area of the target.^[^ ^22^ ^]^ However, their usage is limited by fast excretion and undesirable side effects.^[^ ^23^ ^]^ Therefore, a wide range of research has been focused on the investigation of nanoparticle‐based MRI contrast agents.^[^ ^24^, ^25^ ^]^ Compared with conventional molecular coordination complexes, nanoscale contrast agents show several outstanding advantages, including markedly improved contrast effect, long blood circulation time, and flexible surface chemistry.^[^ ^26^ ^]^ Most currently existing nanoparticulate contrast agents are simple nanoparticles without multilevel microarchitecture,^[^ ^19^, ^27^ ^]^ whose contrast effect can be optimized by regulating their size,^[^ ^28^, ^29^ ^]^ shape,^[^ ^30^, ^31^ ^]^ composition,^[^ ^32^, ^33^, ^34^, ^35^ ^]^ core‐shell structure,^[^ ^36^, ^37^ ^]^ crystallinity,^[^ ^38^, ^39^ ^]^ surface modification,^[^ ^40^, ^41^ ^]^ and assembled structure.^[^ ^42^, ^43^, ^44^ ^]^ Notably, the microhierarchical structure of contrast agents is directly related to the dynamic interactions between water molecules and the magnetic centers, which is an important factor that needs to be manipulated to obtain high‐performance contrast agents. Thus, nano‐metamaterials with tunable microhierarchical structures show great potential for developing highly effective MRI contrast agents (**Table** **1**). In contrast to conventional nanoparticulate contrast agents with single‐level microarchitectures, the unique multilevel microarchitecture of nano‐metamaterials can alter a variety of imaging‐related parameters, thus inducing a coordinative effect to improve the MRI performance (**Figure** **3**). In this regard, we present a detailed description of the superiority of nano‐metamaterials for MRI according to the imaging principles.

**Table 1:** The advantages and limitations of nano‐metamaterials and conventional materials

Contrast agents	Molecular coordination complexes	Nanoparticles	Nano‐metamaterials
Microarchitecture	Simple	Simple	Complex
Precise arrangement of components at nanoscale	No	No	Yes
Blood circulation time	Short	Long	Long
Surface modification	Poor	Flexible	Flexible

Typically, based on the classical Solomon–Bloembergen–Morgan (SBM) theory, the longitudinal relaxivity (*r*_1_) of *T*_1_ contrast agents can be modeled as following^[^ ^45^ ^]^

(1) $$r_{1}=r_{1}^{\text{IS}}+r_{1}^{\text{SS}}+r_{1}^{\text{OS}}\;$$

(2) $$r_{1}^{\text{IS}}=\frac{q/[H_{2}O]}{T_{\text{1m}}+\tau_{m}}$$

(3) $$\frac{1}{\tau_{c}}=\frac{1}{T_{\text{1e}}}+\frac{1}{\tau_{m}}+\frac{1}{\tau_{R}}$$

(4) $$\frac{1}{T_{\text{1m}}}=\frac{2}{15}(\frac{\mu_{0}}{4\pi })\frac{\gamma_{H}^{2}g_{e}^{2}\mu_{B}^{2}S(S+1)}{r_{\text{CH}}^{6}}\left[\frac{{3\tau }_{c}}{{1+\omega }_{H}^{2}\tau_{c}^{2}}\right]$$

As shown in Equation (1), the *r*_1_ value of *T*_1_ contrast agents consists of three portions, including inner sphere relaxivity (*r*_1_^IS^), second sphere relaxivity (*r*_1_^SS^), and outer sphere relaxivity (*r*_1_^OS^), among which, the *r*_1_^IS^ that arises from the water protons directly binding to the paramagnetic ions of *T*_1_ contrast agents is the most important contributor for *r*_1_ value.^[^ ^46^ ^]^ In Equation ((2), (3))–(4), *q* is the number of water molecules in the inner sphere; *T*_1m_ and $\tau_{m}\;$are the *T*_1_ relaxation times and residency times of water protons in the inner sphere; $\tau_{C}$ is the correlation time for describing the fluctuating magnetic dipole; $\tau_{R}$ represents the rotational correlation times of *T*_1_ contrast agents; $r_{\text{CH}}$ is the distance between the metal ions and water molecules; *T*_1e_ describes the electronic *T*_1_ relaxation process; $\mu_{0}$, $\gamma_{H}$, $g_{e}$, $\mu_{B}$, *S*, and $\omega_{H}$ are constants that represent the permeability of the vacuum, the gyromagnetic ratio of the proton, the electric g‐factor, the Bohr magneton, the total electron spin of the paramagnetic ions and the proton Larmor frequency, respectively.

The *T*_1_ contrast effects of conventional nanoparticle‐based contrast agents are mainly attributed to the interactions between paramagnetic ions (e.g., Fe^3+^, Gd^3+^, Mn^2+^, etc.) on nanoparticle surfaces and surrounding water molecules.^[^ ^41^ ^]^ In contrast, nano‐metamaterials with dense multilevel periodic structures can facilitate the access of water molecules to their internal paramagnetic ions and trap water molecules in the interstices of their complicated microarchitectures, endowing them with larger *q* and longer $\tau_{m}$ than that of conventional nanoparticulate contrast agents. Moreover, the immobilization of paramagnetic ions in nano‐metamaterials extremely limits their free rotation, leading to an increase in the $\tau_{R}$. According to Equation ((1), (2), (3))–(4), with large *q* as well as long $\tau_{m}$ and $\tau_{R}$, nano‐metamaterials can significantly accelerate the *T*_1_ relaxation times of water protons, thereby, exhibiting an appreciable *T*_1_ contrast effect. In contrast, the development of superparamagnetic nano‐metamaterials is also desirable for *T*_2_‐weighted MRI. Benefiting from their dense multilevel‐ordered periodic arrangement of the substructure, the overall crystallinity, rigidity, and magnetism, nano‐metamaterials are expected to exhibit a remarkable contrast enhancement compared with previously reported nanosized contrast agents with single‐level microarchitectures, which can intensify the perturbation of nuclear spin relaxation of surrounding water protons under an applied magnetic field,^[^ ^47^ ^]^ thus achieving significantly enhanced *T*_2_ contrast effect.

Apparently, the complex microhierarchical structure of nano‐metamaterials greatly contributes to their MR contrast capability, which can be readily modulated to obtain high‐performance MRI contrast agents. The cutting‐edge nano‐metamaterials with outstanding imaging performance have a broad application prospect in the monitoring of previously undetectable biological entities in living systems.

## 3 MRI Application of Nano‐Metamaterials

**Figure 4:**
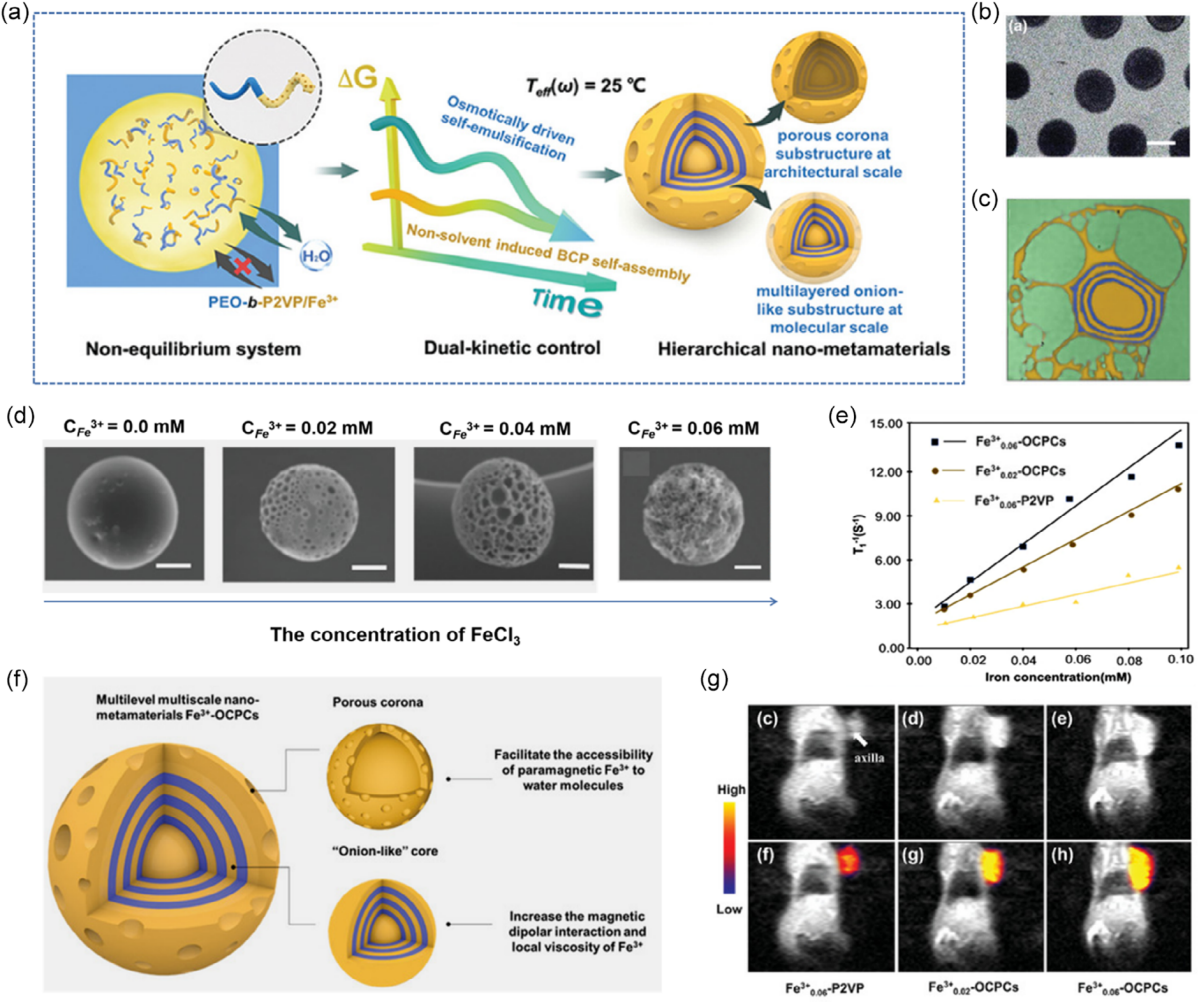
a) Schematic illustration of the fabrication of Fe^3+^–OCPCs based on the novel dual‐kinetic control strategy. b) Transmission electron microscopy (TEM) image of monodisperse Fe^3+^_0.06_–OCPCs; scale bar: 500 nm. c) High‐resolution TEM image of Fe^3+^_0.06_–OCPCs slices (pseudocolor). d) Scanning electron microscopy (SEM) images of Fe^3+^–OCPCs produced at different Fe^3+^ concentrations of 0, 0.02, 0.04, and 0.06 mm, respectively. Scale bar: 100 nm. e) *T*_1_ relaxation rate of water proton at the presence of Fe^3+^_0.06_–OCPCs, Fe^3+^_0.02_–OCPCs, and Fe^3+^_0.06_–P2VP. f) Schematic diagram of the relationship between the microarchitectures of Fe^3+^_0.06_–P2VP nano‐metamaterial and MRI performance. g) In vivo *T*_1_ contrast enhancement of Fe^3+^_0.06_–P2VP, Fe^3+^_0.02_–OCPCs, and Fe^3+^_0.06_–OCPCs in mice with subcutaneous axillary tumors after 30 min intratumor injection. a–g) Reproduced under the terms of the CC‐BY Creative Commons Attribution 4.0 International license (https://creativecommons.org/licenses/by/4.0).^[^ ^52^ ^]^ Copyright 2023, The Authors, Wiley‐VCH.

Metamaterials with multilayered and multilevel microstructures exhibit distinctive properties that are progressively applied in biomedical fields, including wearable stretchable sensors,^[^ ^48^ ^]^ and transdermal drug delivery systems.^[^ ^49^ ^]^ However, the synthetic limitations, such as size control, remain significant challenges for the development of well‐defined nano‐metamaterials as cutting‐edge imaging probes for biomedical imaging applications. As a result, despite nano‐metamaterial is theoretically a highly promising candidate for MRI, practical MRI applications using nano‐metamaterial are extremely scarce. Indeed, conventional nanomaterials with single‐level microarchitectures follow the law of minimum energy,^[^ ^50^ ^]^ while the construction of hierarchical nano‐metamaterials is relatively challenging from the point of thermodynamics. A system in thermodynamic equilibrium has a constant frequency‐dependent effective temperature (*T*_eff_(*ω*)), where deviation from the *T*_eff_(*ω*) will lead to the generation of a new structure with a new equilibrium state.^[^ ^51^ ^]^ However, the thermodynamic process only depends on the *T*_eff_(*ω*), which limits the freedom degrees of architectural regulation.^[^ ^52^ ^]^ In contrast, the time‐dependent dynamic pathway owns multiple variable parameters, providing opportunities for creating various nonequilibrium structures under a *T*_eff_(*ω*)‐constant system.^[^ ^53^ ^]^ Accordingly, Ling, Wang, and co‐workers^[^ ^52^ ^]^ proposed a pioneering dual‐kinetic control strategy to fabricate multilevel multiscale nano‐metamaterials by manipulating dynamic processes in a thermodynamically constant system, where the two independent kinetic pathways, nonsolvent‐induced block copolymer (BCP) self‐assembly and osmotically driven self‐emulsification could be simultaneously regulated (**Figure** **4**a). Utilizing this strategy, multilevel multiscale Fe^3+^–“onion‐like core porous crown” nanoparticles (Fe^3+^–OCPCs), which consist of two substructures: 1) an onion‐like core; and 2) a hierarchical porous corona, were successfully prepared (Figure 4b,c). Notably, the number and size of the pores in the hierarchical microarchitectures of Fe^3+^–OCPCs can be precisely controlled by changing the concentration of Fe^3+^ (Figure 4d). Moreover, as shown in Figure 4e, the Fe_0.02_^3+^–OCPCs and Fe_0.06_^3+^–OCPCs exhibited an excellent *T*_1_ contrast effect with *r*_1_ values of 10.48 and 13.39 mm ^−1^ s^−1^, respectively, which were 2.5‐ and 3.4‐fold higher than that of homogeneous Fe_0.06_^3+^–poly(2‐vinylpyridine) (P2VP) nanoparticles (4.21 mm ^−1^ s^−1^, 3.0 T), primarily attributed to the distinctive hierarchically ordered multilevel structure of Fe^3+^–OCPCs. On one hand, the hierarchical porous corona facilitates the sufficient contact between paramagnetic Fe^3+^ and surrounding water protons, which is conducive to promoting the *T*_1_ relaxation of water protons. On the other hand, the onion‐like core accommodated multilayers of block polymer can increase the magnetic dipolar interaction and local viscosity of Fe^3+^, thus prolonging the $\tau_{m}$ of Fe^3+^–OCPCs by limiting the mobility of water molecules nearby paramagnetic Fe^3+^ (Figure 4f). Accordingly, compared with homogeneous Fe^3+^–P2VP, Fe^3+^–OCPCs exhibited superior *T*_1_ contrast enhancement effect in vivo and effectively light up tumors 30 min after injection (Figure 4g).

Therefore, such a dual‐kinetic control strategy with two independent dynamic processes represents an effective strategy for constructing well‐defined nano‐metamaterials, which serves as a proof‐of‐concept to demonstrate that the architectural regulation of nanoparticles is important for MRI performance. Nevertheless, the colloid stability of current nano‐metamaterials in biological environments needs to be further improved. Moreover, the precise size control of nano‐metamaterials is imperative for broader MRI applications in living systems.

## 4 Future Perspective

Owing to the multilevel‐ordered microarchitectures, nano‐metamaterials hold great potential in developing next‐generation high‐performance MRI contrast agents, which is attributed to their capability to enhance the important parameters affecting the relaxation times of water protons, such as hydration number, rotational correlation times, water residence times, magnetic perturbation, and potential others. However, one should be aware that the development of nano‐metamaterials as MRI contrast agents is still in the proof‐of‐concept stage, and several challenges remain to be addressed before further expanding their biomedical imaging applications.

First, the precise control and regulation of the size, multilevel microarchitecture, and magnetic property of nano‐metamaterials are urgently needed to ensure the tunable MRI performance. Especially, controllable large‐scale production of nano‐metamaterials with a maximum size down to <100 nm is necessary for in vivo delivery and imaging. Therefore, more efforts should be devoted to optimizing nano‐metamaterials based on the dual‐kinetic strategy via adjusting reaction conditions, such as *T*_eff_(*ω*), solvents, and reactants. Besides, the further development of other advanced synthetic approaches for the controllable fabrication of nano‐metamaterials is highly desired. Second, the surface ligands of nano‐metamaterials can directly influence their microarchitectures, colloidal stability, and disease‐targeting capability. Since the complex biological environments would greatly affect the biological fates of nano‐metamaterials, the increasing understanding of nano–bio interactions and the optimization of the surface modification are extremely helpful to obtain highly efficient nano‐metamaterials for in vivo imaging. Third, to develop a new class of diagnostic agents based on nano‐metamaterials with multilevel multiscale microarchitectures, the relationship among the synthesis parameters, microarchitectures, and biological properties of nano‐metamaterials has to be comprehensively and systematically studied, which is expected to provide a valuable theoretical basis for extensive biomedical applications. Additionally, the ultimate goal of biomedical imaging is to provide guidance for further disease treatment. Accordingly, various therapeutic agents, such as functional nanoparticles, molecular drugs, and bioactive proteins, can be introduced into the layered microstructure of nano‐metamaterials to construct multifunctional systems with integrated imaging and therapeutic functions, for high‐performance imaging‐guided therapy of major diseases, including malignant tumors, cardiovascular diseases, and neurodegenerative diseases.

All in all, the multilevel, multiscale, and complexity exhibited by the microarchitecture of nano‐metamaterials endow them with significant superiority as next‐generation MRI contrast agents. We anticipate the ingeniously designed high‐performance nano‐metamaterials shall greatly promote groundbreaking medical diagnosis research and elucidate unknown life phenomena, spearheading the new era of 21st‐century precision medicine.

## Conflict of Interest

The authors declare no conflict of interest.
